# Formation of Iron Sulfides on Carbon Steel in a Specific Cement Grout Designed for Radioactive Waste Repository and Associated Corrosion Mechanisms

**DOI:** 10.3390/ma14133563

**Published:** 2021-06-25

**Authors:** Mathieu Robineau, Valérie Deydier, Didier Crusset, Alexandre Bellefleur, Delphine Neff, Enrique Vega, René Sabot, Marc Jeannin, Philippe Refait

**Affiliations:** 1 Laboratory of Engineering Sciences for the Environment (LaSIE), UMR 7356 CNRS-La Rochelle University, Av. Michel Crépeau, CEDEX 01, F-17042 La Rochelle, France; mathieu.robineau@univ-lr.fr (M.R.); rsabot@univ-lr.fr (R.S.); mjeannin@univ-lr.fr (M.J.); 2 Research and Development Department, Andra, Parc de la Croix Blanche, 1/7 Rue Jean Monnet, F-92298 Châtenay-Malabry, France; valerie.deydier@andra.fr (V.D.); didier.crusset@andra.fr (D.C.); 3 EDF Lab, Avenue des Renardières, 77250 Écuelles, France; alexandre.bellefleur@edf.fr; 4 Laboratoire Archéomatériaux et Prévision de l’Altération (LAPA) NIMBE–IRAMAT, CEA/CNRS, Université Paris Saclay, UMR 3685, CEA Saclay, Bat 637, 91191 Gif/Yvette, France; delphine.neff@cea.fr (D.N.); enrique.vega@cea.fr (E.V.)

**Keywords:** carbon steel, Raman spectroscopy, X-ray diffraction, SEM, iron sulfide, interfaces

## Abstract

Carbon steel coupons were buried in a specific low-pH cement grout designed for radioactive waste disposal and left 6 months in anoxic conditions at 80 °C. The corrosion product layers were analyzed by µ-Raman spectroscopy, XRD, and SEM. They proved to be mainly composed of iron sulfides, with magnetite as a minor phase, mixed with components of the grout. Average corrosion rates were estimated by weight loss measurements between 3 and 6 µm yr^−1^. Corrosion profiles revealed local degradations with a depth up to 10 µm. It is assumed that the heterogeneity of the corrosion product layer, mainly composed of conductive compounds (FeS, Fe_3_S_4_, and Fe_3_O_4_), promotes the persistence of corrosion cells that may lead to locally aggravated degradations of the metal. New cement grouts, characterized by a slightly higher pH and a lower sulfide concentration, should then be designed for the considered application.

## 1. Introduction

**Figure 1:**
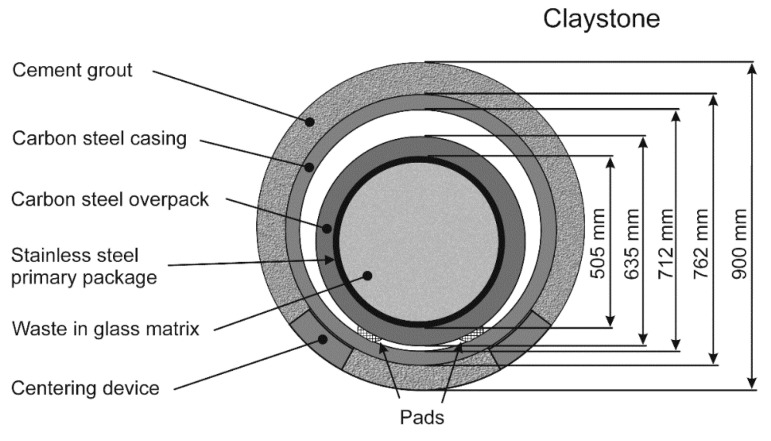
The envisioned multi-barrier concept for the French disposal of high-level radioactive waste: cross-sectional view.

In France, via the Cigéo project [1], there is a plan to dispose of high-level and intermediate-level long-lived radioactive waste in a deep geological disposal. This disposal is to be set at a depth of ~500 m in the Callovo-Oxfordian (Cox) claystone, i.e., a stable 145-m-thick clay formation with very low permeability. For that purpose, a carbon steel casing will be inserted inside horizontal tunnels (“disposal cells”) drilled in the Cox claystone. The carbon steel containers filled with a stainless-steel primary package containing vitrified radioactive waste will be equipped with pads and slipped into the steel casing previously inserted inside the disposal cells. A centering device should avoid any contact between the base of the carbon steel casing and the claystone (Figure 1). The steel surface should reach a temperature as high as 90 °C because of the radioactivity. Once filled with containers, the disposal cell will be sealed, and the oxygen trapped in the system should be consumed more or less rapidly by the corrosion of carbon steel and by other chemical processes occurring in the claystone.

One of the technical specifications considered for the Cigéo project is to inject a specific cement grout between the carbon steel casing and the claystone. The cement grout tested in this study has been designed by Andra according to particular specifications. The most important point is that the cement grout must be able to neutralize any acidity due to the oxidation of the sulfur-containing species present in the claystone, e.g., pyrite [2,3], after drilling operations. However, another specific point is that the pH must not be too basic, to avoid damaging the glass matrix confining the radioactive waste. Actually, if the temperature would be higher than 50 °C when the pore solution reaches the glass, through completely corroded regions of the steel containers, the glass matrix may suffer some significant degradation if the pH were too high. Thus, the specific cement grout was designed to have a pore solution with a pH of 10.7 at 20 °C, which is considered as a “low” pH for cementitious materials.

To reach such a “low” pH, the Portland cement (denoted by the abbreviation OPC or CEM I following European standard EN 197-1) with a pH value of 13 is partially replaced with other materials such as fly ash, blast furnace slag, or silica fume. Thus, in contrast to the generally used CEM I cement that contains at least 95% clinker, the CEM III/C cement used in this study contains between 5 and 19% clinker and a minimum of 81% blast furnace slag (according to European standard EN 197-1). This cement is suitable for making mortars/injection grouts in soils, with low alkalinity and limited heat of hydration. When mixed with this cement, the silica fume, acting as a pozzolanic addition, participates in limiting the alkalinity of the material. It is thus generally used for the formulation of low-pH concretes.

Little information is available on the corrosion of carbon steel buried in cementitious materials with a pH in the range of 10–11. Most studies focused on Portland cement with a pH of about 13. It is widely accepted that carbon steel used as reinforcement in concrete is in a “passive” state, i.e., covered with a protective oxide layer, leading to very low corrosion rates (≤0.1 µm yr^−1^) [4,5,6,7,8]. Borgard et al. established the concept of passivity of reinforcing steel in concrete, according to potential-pH (Pourbaix) diagrams [9]. The corrosion of the reinforcement can be due to two main phenomena [10]: the carbonation of the concrete which induces general corrosion and the penetration of chloride which produces local depassivation of the reinforcement at a certain chloride concentration threshold. Many articles were published to determine the critical value of this threshold, and it was finally proposed that it depends on the Cl^−^ to OH^−^ concentration ratio [10]. Glass [11] and Andrade [12,13] studied the effect of this ratio on the corrosion processes of steel in a synthetic concrete pore solution. They both concluded that the Cl^−^/OH^−^ concentration ratio was indeed the predominant chemical bulk parameter controlling the corrosion rate.

In order to estimate the transition pH between passive and active behavior of steel in contact with concrete, Huet et al. [14] conducted immersion tests with five electrolytes. These electrolytes, with a pH ranging from 8.3 to 13, were chosen to simulate pore water in concrete at different levels of carbonation. The results indicated that the transition pH was between 9.4 and 10. XPS analysis indicated a passivation of steel for pH values between 10 and 13 due to the formation of a very thin oxide layer (<10 nm) mainly composed of Fe(III) oxides. Decreasing the pH and/or carbonate concentration of the electrolyte promoted active corrosion of the steel sample, resulting in the formation of a large amount of a Fe(II) compound that did not protect the steel substrate from further corrosion.

Only one preliminary study dealing with the corrosion of carbon steel in the specific cement grout envisaged for radioactive waste disposal was achieved [15]. In this work, carbon steel electrodes covered by a 2-cm-thick layer of the grout were immersed in a 0.01 M NaCl + 0.01 M NaHCO_3_ solution (pH 7 measured at 20 °C) at 80 °C in aerated conditions. A passive to active transition was observed in a few days, indicating that in these experimental conditions the metal could not remain passive for a long time [15]. It was also shown that iron sulfides could form as corrosion products on the steel surface. This result was attributed to the small amount of sulfide species present in the cement grout and coming mainly from the CEM III/C cement (0.77 wt.%).

This preliminary study clearly showed that the corrosion processes in the specific cement grout and in the particular conditions of the radioactive waste disposal were significantly different from those commonly involved for steel in Portland cement and concrete. The unexpected iron sulfides and their role in the corrosion process needed in particular to be more specifically addressed. For that purpose, the present study was performed in conditions very close to those expected in the Cigéo site, i.e., in anoxic environment at 80 °C and using the solution expected to fill the pores of the fresh cement grout. Moreover, longer experiments (6 months) were carried out. To obtain information about the role of iron sulfides and associated corrosion mechanisms, a detailed analysis of the corrosion product layer was achieved using X-ray diffraction (XRD), µ-Raman spectroscopy (µ-RS), and scanning electron microscopy (SEM). Average corrosion rates were determined by weight loss measurements, and local degradations were investigated by optical profilometry measurements performed on the steel surface after removal of the corrosion product layer.

## 2. Materials and Methods

### 2.1. Materials and Electrolytes

The material tested was API 5L X65 carbon steel, prepared as rectangular coupons having an active surface of 18.4 cm^2^. API 5L X65 is the alloy retained for the casing to be inserted inside the tunnels of the radioactive waste disposal (see Figure 1). Its nominal composition in weight % is given in Table 1. The surface of the coupons was abraded with silicon carbide (grade 600, particle size 25 µm), rinsed with deionized water, and kept in a desiccator before the beginning of the experiment.

**Figure 2:**
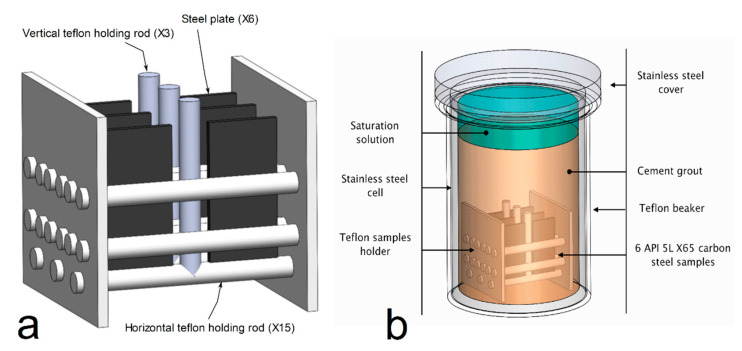
Schematic representation of (**a**) the Teflon sample holder with 6 carbon steel samples and (**b**) schematic view of the test cell.

The specific grout was prepared in air by mixing the different components listed in Table 2. Evian water was used preferentially to tap water because it has a more controlled composition and a low chloride concentration of 1.3 × 10^−4^ mol/L. After the different stages of its preparation, the grout was poured in a Teflon beaker itself placed in a stainless-steel cell. A Teflon holder was used to maintain 6 carbon steel coupons in the Teflon beaker as illustrated in Figure 2. The use of Teflon rods in the design of the sample holder (Figure 2a) reduces the contact area between API 5L X65 steel specimens and Teflon. The vertical holding rods between the specimens in the same row provides a better hold.

Figure 2b displays a schematic view of the device used for this study. The device consists of a stainless-steel cell with a Teflon beaker inside and a stainless-steel cover. The sealing between the edge of the Teflon beaker and the bottom of the cover is made with a nitrile seal resistant to alkaline solutions at 80 °C. The sample holder containing the steel coupons and the cement grout are set inside the Teflon beaker.

After closing the stainless-steel cell, an argon flow was flushed during 2 h to remove oxygen from the cell and reach anoxic conditions. The cement grout was kept 8 weeks in a controlled argon atmosphere at room temperature (RT = 20 ± 2 °C). This initial 8-week stage is required for the cement grout to harden and reach its final state (for that reason, the cement grout should be injected between the steel casing and the Cox claystone at least 8 weeks before the arrivals of the radioactive waste packages). The pH of the cement grout solution was measured at 20 °C using the procedure described in the NF ISO 10390 standard [16]. A value of 10.7 ± 0.1 was obtained at the end of the initial 8-week period. After this period, a solution was introduced in the cells to simulate the pore water reached in the cement grout at the first stages of the storage. The addition of the solution was performed in argon atmosphere inside a glove box. The cell was finally placed in an oven at 80 °C where it remained for 6 months.

The composition of the solution, provided by Andra (Châtenay-Malabry, France) for the present study, is given in Table 3. It was obtained from numerical chemical simulations of the equilibrium conditions of the pore electrolyte/cement grout system. It can be seen that the main anionic species present in the solution are sulfate and carbonate. According to a specific procedure based on these chemical calculations, the pH of the solution had to be adjusted at 50 °C to a value of 10.5 ± 0.1 with a solution of 1 M NaOH.

### 2.2. µ-Raman Spectroscopy, X-ray Diffraction, and Scanning Electron Microscope Analysis of Corrosion Product Layers

At the end of the experiments, the cement grout surrounding the steel samples was removed so that the analysis of the corrosion products could be performed. In any case, the corrosion product layer adhered to the steel surface, and it is assumed that only a minor part of the layer was possibly removed with the grout.

µRS was performed with a Jobin Yvon High Resolution Raman spectrometer (LabRAM HR, Horiba, Tokyo, Japan) equipped with a Peltier-based cooled charge-coupled device detector. The analyzed zones had a diameter of ~3 µm and were observed at a magnification of 50× through the microscope (Olympus BX 41, Olympus, Tokyo, Japan) of the Raman apparatus. The spectra were recorded with the acquisition LabSpec software at RT with a resolution of 0.4 cm^−1^. Excitation was provided by a He-Ne laser (wavelength of 632.8 nm). Its power was limited to 0.9 mW, which is 10% of the maximal power, in order to prevent an excessive heating that could induce the transformation of the analyzed compounds into hematite. The investigations were performed without specific protection from air. The acquisition time was then generally short (30 s^−1^ min) to minimize the risk of transformation of the analyzed compounds by oxidation and/or heating. The corrosion product layer present on each side of the sample was characterized via at least 40 Raman spectra. This was necessary as µRS analysis is a local characterization technique whereas the corrosion product layers proved very heterogeneous.

XRD analysis was performed with an Inel EQUINOX 6000 diffractometer (Thermo Fisher Scientific, Waltham, MA, USA), using the curved detector CPS 590, with a cobalt X-ray source (CoKα wavelength = 0.17903 nm). The curved detector detects simultaneously the diffracted photons on a 2*θ* range of 90°. Acquisition was made with a constant incidence angle of 5 degrees during 45 min. To prevent the oxidation of the corrosion products during preparation and analysis, the surface of the corrosion product layer was covered with glycerol. This liquid phase only gives rise to a very broad “hump” visible on the XRD pattern between 2*θ* = 17° and 2*θ* = 30°.

SEM analysis was performed with a JEOL JSM-7001F (JEOL Ltd., Tokyo, Japan) coupled with an 80 mm^2^ X-ray silicon drift detector associated with an energy dispersive spectrometer (EDS) from Oxford Instruments PLC (Abigdon-on-Thames, UK). The electron acceleration voltage was 15 kV, the probe current 5 nA, and the working distance was 10 mm. A metallization of the surfaces by carbon deposition (20 nm thick) was performed prior to the analysis. SEM was used to observe both the surface topography of the corrosion product layer and the layers’ cross-section. In the case of surface topography, the cement grout was removed just before the sample was set inside the SEM analysis chamber. In the case of cross-sectional analysis, the system composed of steel, corrosion product layer, and grout was placed in epoxy resin before being polished with silicon carbide paper in the presence of heptane to avoid oxidation of the Fe(II) compounds possibly present in the corrosion product layer. Images of the products covering the steel surface were taken with the secondary electrons detector to access topographic information. Phi-Rho-Z spectra treatment processed by Aztec software (Oxford Instruments) was used to quantify the various elements of interest, i.e., Fe, S, O, Ca, Al, and Si. Elemental hypermapping was carried out to observe the distribution of the elements on both the surface and cross-sections of the system.

Two samples were analyzed by µRS and XRD, two others by SEM-EDS. The two samples analyzed by µRS and XRD were also used to quantify the degradation. Average corrosion rates were determined via weight loss measurements and localized corrosion rates via optical profilometry measurements.

### 2.3. Weight Loss Measurements and Corrosion Depth Profiles

The method used to remove the corrosion product layers from the steel coupons was based on the ASTM G1 standard [17]. After removal of the cement grout, the coupons were immersed in a solution composed of hydrochloric acid (500 mL), hexamethylenetetramine (3.5 g), and deionized water (500 mL). The corrosion rate was then calculated according to Equation (1) given in ASTM G1:

(1) $$\mathrm{Corrosion}\;\mathrm{rate}=\frac{\left(K\times W\right)}{\left(A\times T\times D\right)}$$

with *K*: a constant (8.76 × 10^4^ to have a corrosion rate in mm yr^−1^), *T*: the exposure time in hour (4320 h), *A*: surface in cm^2^ (18.4 cm^2^), *W*: weight loss in g (initial weight–weight after desquamation), and *D*: density in g cm^−3^ (iron: 7.86 g cm^−3^).

In addition, corrosion depth profiles and quantification of local corrosion damages were obtained using a Fogale optical profilometer. The interferometric sensor has a vertical resolution of 3.0 nm. Measurements were carried out in the areas that appear, after visual observations, to be the most degraded. They were performed with a Z-step of 10 nm, and the length of the profile was chosen as defined in the NF EN ISO 4287 standard [18]. Note that profilometry measurements were not carried out in the zones where the carbon steel was in contact with rods of the Teflon holder, where specific processes due to side effects (e.g., crevice corrosion) may have taken place.

## 3. Experimental Results

### 3.1. Preliminary Analysis

First, to investigate the real impact of the test solution and the increase of temperature to 80 °C, studies related to the effects of the preliminary 8-week period at RT under controlled Ar atmosphere were needed. The surface of three coupons, subsequently not used for the corrosion study, was characterized after this period by optical microscopy observation and µRS. In each case, the steel surface appeared undamaged and µRS analysis did not reveal the presence of corrosion products. In fact, it was still possible to see the polishing grooves due to the initial surface preparation. Weight loss measurements performed on the three coupons confirmed that the degradation (if any) was negligible. The steel coupons more likely remained in a passive state during the 8 weeks of setting at RT, as observed in a previous study [15] achieved in aerated conditions.

### 3.2. µRS and XRD Analysis

**Figure 3:**
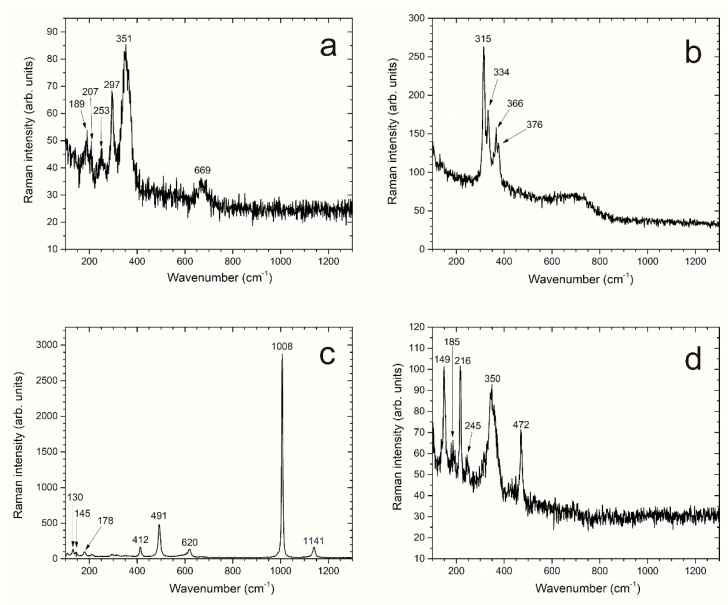
Typical Raman spectra obtained during the analysis of carbon steel samples after the experiment. (**a**) Greigite/mackinawite/magnetite mixture; (**b**) unknown compound; (**c**) gypsum; (**d**) greigite/elemental sulfur.

Typical Raman spectra of the samples after the 6-month experiment are displayed as examples in Figure 3. The Raman spectrum of Figure 3a presents the spectral components of three corrosion products, i.e., magnetite (Fe_3_O_4_), greigite (Fe_3_S_4_), and mackinawite (FeS). Magnetite is identified via its characteristic main Raman peak at 669 cm^−1^ [19], greigite is identified via the Raman peaks at 189 cm^−1^ and 351 cm^−1^ [20], and mackinawite via the Raman peaks at 207 cm^−1^, 253 cm^−1^, and 297 cm^−1^ [21].

Another corrosion product was frequently observed. However, its Raman signature does not correspond to any spectrum listed in the literature (Figure 3b). Its main peak at 315 cm^−1^ and the other peaks at 334 cm^−1^, 366 cm^−1^, and 376 cm^−1^ suggest that it could be an iron sulfide with a chemical formula close to that of pyrite and marcasite (FeS_2_). The Raman spectra of both FeS_2_ compounds display peaks in the same spectral domain, more precisely at 343 cm^−1^ and 379 cm^−1^ for pyrite and 323 cm^−1^ and 386 cm^−1^ for marcasite [22].

**Figure 4:**
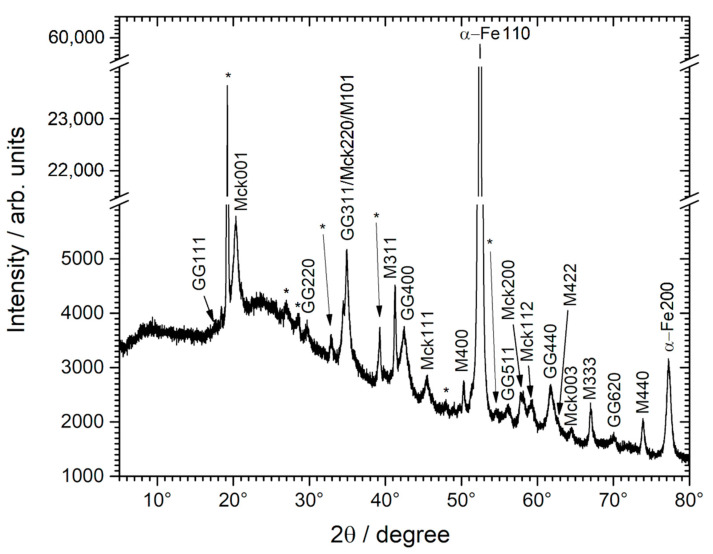
XRD analysis of the corrosion product layer covering a carbon steel sample after the experiment. GG, Mck, M and, α-Fehkl are the diffraction peaks of greigite, mackinawite, magnetite, and α-Fe with the corresponding Miller index. The * points out the diffraction peaks of an unidentified corrosion product.

XRD analysis, illustrated by the pattern shown in Figure 4, confirmed the presence of greigite, mackinawite, and magnetite. As with µ-Raman spectroscopy, some peaks could not be attributed. They may correspond to the unknown compound suspected to be an iron sulfide. However, they do not correspond to marcasite nor pyrite (International Centre for Diffraction Data “ICDD”—Joint Committee on Powder Diffraction Standards “JCPDS” file 01-073-8127 for pyrite and 03-065-2567 for marcasite), although Raman analysis suggested some similarity between this unknown corrosion product and the FeS_2_ phases.

Other compounds were more rarely found on the steel surface, namely Fe(III)-containing mackinawite (Fe^II^_1−3x_Fe^III^_2x_S, data not shown), calcium sulfates (gypsum in Figure 3c and anhydrite, not shown), and elemental sulfur *α*-S_8_ associated with greigite (Figure 3d). Fe(III)-containing mackinawite, greigite, and elemental sulfur can all result from the oxidation of mackinawite (FeS) [20,21]. Gypsum, a hydrated calcium sulfate with formula CaSO_4_·2H_2_O, and anhydrite, an anhydrous form of CaSO_4_, are common minerals. Gypsum is used as an additive for cement and, consequently, both calcium sulfates more likely come from the cement grout. However, their formation may have been favored by the considered solution, which contains calcium and sulfate species (Section 2.1). In Figure 3d, elemental sulfur α-S_8_ is identified via its characteristic Raman peaks at 149, 216, and 472 cm^−1^ [23] and greigite via its peaks at 185, 245, and 350 cm^−1^ [20].

In conclusion, iron sulfides were the main corrosion products identified, particularly greigite and mackinawite ones. Magnetite was also identified as a minor component, detected only locally during µRS analysis. The corrosion product layer can then be described as a heterogeneous layer mainly made of iron sulfides with scattered zones containing magnetite. Because the solution used to saturate the cement grout did not contain sulfide species, these species necessarily come from the grout. This result is consistent with those observed in previous study [15].

### 3.3. Weight Loss and Profilometry Measurements

**Figure 5:**
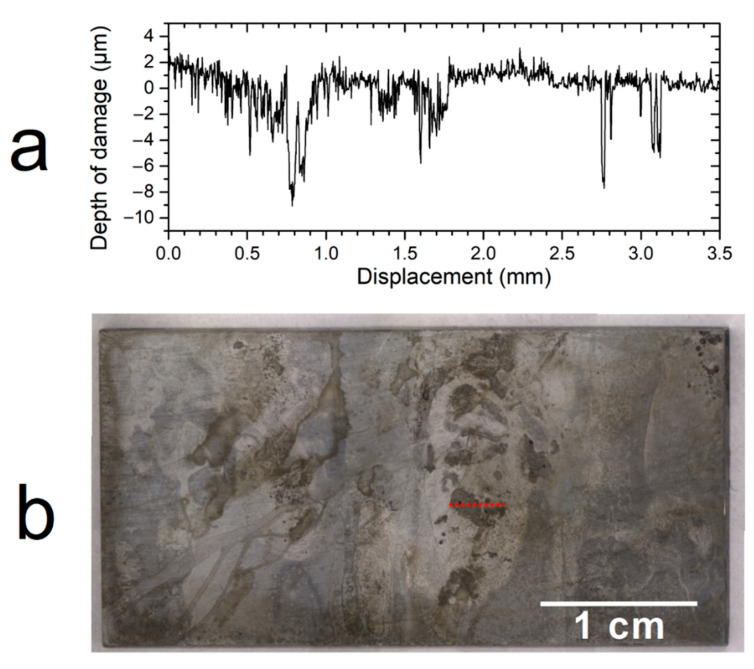
(**a**) Corrosion depth profile obtained in the most degraded area of a carbon steel sample after the experiment and (**b**) image of the coupon surface showing the localization of the analyzed area (red dotted line).

Weight loss measurements led to average corrosion rates of 6 µm yr^−1^ for the first coupon and 3 µm yr^−1^ for the second one. Profilometry measurements performed on the three zones of each coupon clearly revealed the presence, in various areas, of localized degradations. An example of the obtained depth profiles is shown in Figure 5a. In this graph, the origin of the vertical axis actually corresponds to a particular area of the metal surface that appeared moderately corroded. Consequently, less corroded areas appear as positive points (left of the figure). The profilometry study was focused on the areas that seemed, via visual observation, the most severely corroded. The “displacement” axis corresponds to the direction along which the degradation profile was acquired in the considered region of the surface, as shown in Figure 5b.

In the apparently more severely corroded areas, the corrosion depth reached 10.5 µm (in pits having a width between 20 and 50 µm), corresponding to local corrosion rates of 21 µm yr^−1^. The average surface roughness Ra in this analyzed area was evaluated at 0.920 µm. In a previous study [15], localized corrosion phenomena were also observed. The corrosion rates, determined by electrochemical measurements, were significantly higher, between 16 µm yr^−1^ and 250 µm yr^−1^, more likely because of aerated conditions and a higher Cl^−^ concentration.

### 3.4. Characterization by SEM-EDS

Two carbon steel coupons were analyzed using SEM-EDS to observe the surface topography of the corrosion product layer (coupon 1), and its possible stratification via a cross-sectional chemical analysis (coupon 2).

#### 3.4.1. Surface Topography

After removal of the cement grout, several areas of the mineral layer covering the steel coupon were observed and analyzed. The results obtained for two characteristic zones are detailed in the following.

**Figure 6:**
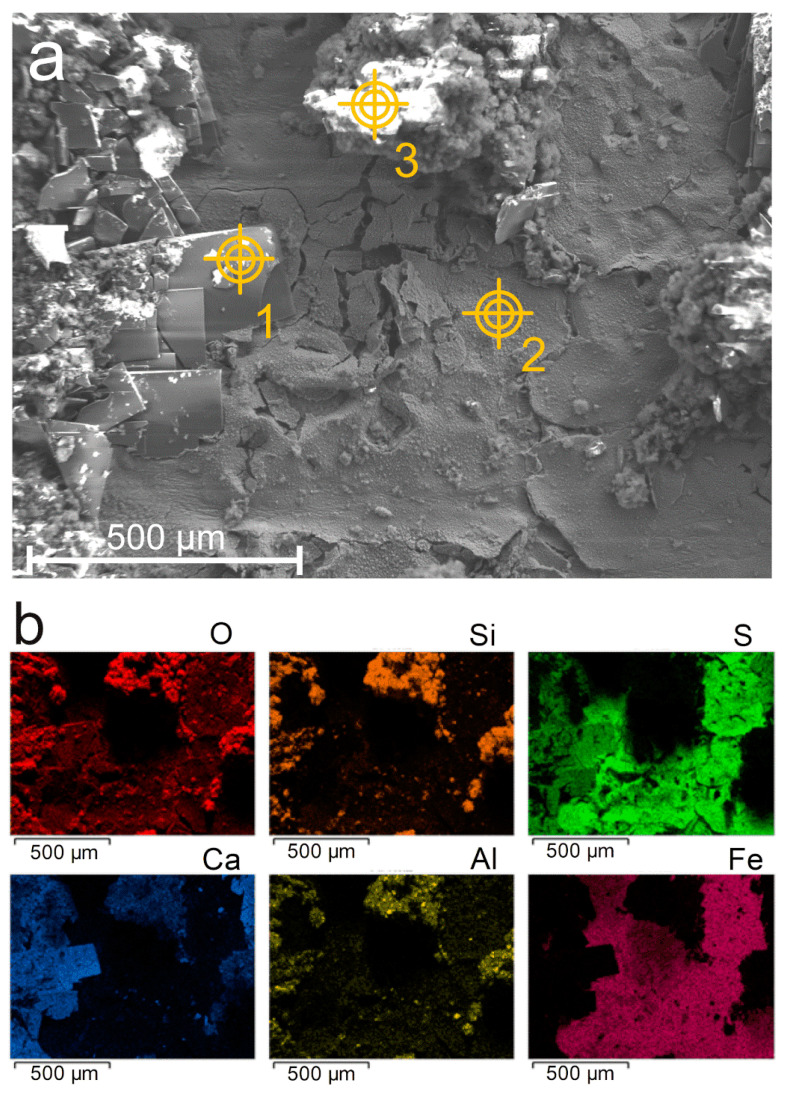
SEM analysis of the surface of the layer covering a carbon steel surface after the experiment. (**a**) SEM image (secondary electrons) of the layer covering the carbon steel surface with 3 spots indicating where quantitative EDS analysis was performed; (**b**) corresponding EDS maps.

Figure 6 relates to the first zone. The SEM image of the surface (Figure 6a) shows that the mineral layer is heterogeneous. Large rectangular particles can be seen (1) together with conglomerates of small spherical particles (3) that seem somehow deposited on a smoother and more homogeneous, though cracked, underlying layer (2).

The corresponding EDS maps are displayed in Figure 6b. They show that the compounds present in the outer parts of the layer, i.e., the rectangular and spherical particles, are mainly constituted of calcium or silicon. Therefore, these particles come from the grout, which explains why they form the outer part of the mineral layer covering the steel surface. In contrast, the underlying layer contains mainly iron and sulfur, and thus corresponds to the corrosion product layer. This result is consistent with µRS analysis, which showed that iron sulfides were the main corrosion products. Additional analysis was performed in the three spots defined in Figure 6a by the yellow targets. The rectangular particles (spot 1) are mainly composed of oxygen, sulfur, and calcium. µRS analysis revealed the presence of calcium sulfates (CaSO_4_) such as gypsum and anhydrite. In addition, this type of morphology was already described in studies of plaster containing crushed gypsum [24], but also more recently in the study of gypsum transformation as a function of temperature [25]. Therefore, the rectangular particles found locally on the steel surface definitely correspond to calcium sulfates. The analysis of the second zone (spot 2) corresponding to the inner part of the layer, i.e., the corrosion product layer, confirms that Fe and S elements are predominant, i.e., that iron sulfides are the main corrosion products. The last analysis (spot 3) relates to a conglomerate of spherical particles. They prove to be mainly composed of elements from the grout (silicon, calcium, and aluminum), and are thus particles of the grout that have adhered to the underlying corrosion product layer.

**Figure 7:**
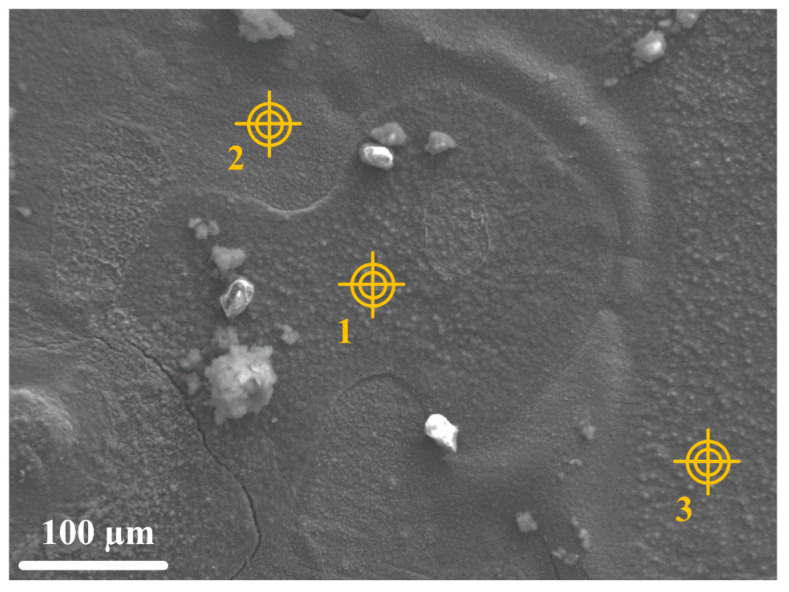
SEM image (secondary electrons) of the surface of the mineral layer present on a carbon steel surface after the experiment. The 3 targets indicate the area analyzed by EDS.

A second characteristic zone was analyzed. The corresponding SEM image is shown in Figure 7. In this area, various particles are scattered over an underlying layer. This layer is composed of a smooth part (spot 2) and a rougher part (spots 1 and 3). Elemental maps (not presented) showed that the smooth area was mainly composed of sulfur and iron, while the rougher zone was composed of sulfur, iron, and silicon. Local elemental analysis was carried out on the three zones indicated by yellow targets. zones 1 and 3 are characterized by a higher content of silicon and oxygen than zone 2, as shown in Table 4. The corrosion product layer covering the surface of the steel is nevertheless, in accordance with µRS analysis, mainly composed of iron sulfides (Fe and S elements). The silicon and oxygen detected at spots 1 and 3 are probably originating from constituents of the grout intricately mixed with the corrosion products. It could be silica fume.

#### 3.4.2. Cross-Sectional Analysis

**Figure 8:**
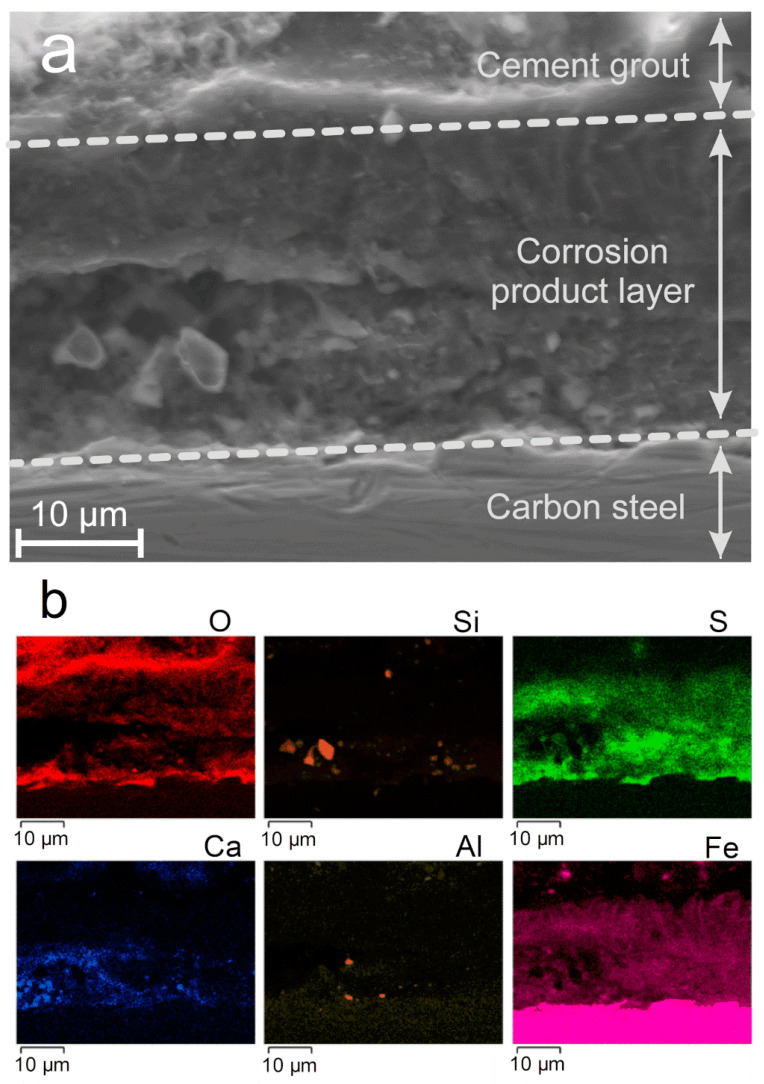
SEM image (secondary electrons) of the carbon steel/corrosion products layer/grout system (cross-sectional view) after the experiment (**a**) and corresponding EDS maps (**b**).

Cross sections were studied to highlight a possible stratification. A SEM image is shown as an example in Figure 8a, and the associated elemental maps are presented in Figure 8b.

This analysis clearly reveals the metal that appears as a bright pink strip at the bottom of the Fe map. It is covered with a corrosion product layer mainly composed of iron, sulfur, and oxygen. Various particles with a different composition are however incorporated in this layer, some of them detected close to the metal surface. They appear as large Si-based particles and smaller Ca-based and Al-based particles. Fe is not present in these particles and consequently they do not correspond to corrosion products. Fe-rich phyllosilicates, which were for instance obtained on steel in bentonite at 120 °C [26], did not form in the experimental conditions considered here. The thickness of the corrosion product layer can be estimated from the Fe and S maps at about 20 µm.

At the steel/corrosion product layer interface, Fe, S, and O are associated inside a compact inner stratum that has a thickness of ~5 µm. It could be composed of a mixture of iron oxides and iron sulfides. This is consistent with the identification, by both XRD and µRS, of magnetite together with the predominant iron sulfides. Above this first stratum is found a second one, depleted in oxygen, which may then be only or predominantly composed of iron sulfides. It is also important to note that in the entire area described here, calcium was identified within the corrosion product layer. It may come from the CaSO_4_ phases present in the grout, as revealed by surface analysis (Figure 6). Finally, particles containing silicon and aluminum are seen inside the corrosion product layer. As they do not contain Fe, these particles probably come from the grout.

## 4. Discussion

The first study devoted to the behavior of carbon steel in the specific cement grout designed for radioactive waste repository was performed in aerated conditions and a more “aggressive” 0.01 mol L^−1^ NaHCO_3_ + 0.01 mol L^−1^ NaCl solution was used [15]. This solution was thus characterized by a much larger Cl^−^ concentration than the solution considered here, and its pH was equal to 7, which was likely to favor a decrease of pH inside the grout. As a result, it was observed that steel did not remain passive more than a few days once the system was heated to 80 °C [15]. The experimental conditions of the present study are significantly different: low Cl^−^ concentration, pH of the solution equal to 10.5, anoxic conditions, and higher cement grout to solution volume ratio. However, the passive state did not persist even in this case and a corrosion process took place, leading to a corrosion product layer mainly composed of iron sulfides.

As already observed in [15], the steel is however passive at room temperature during the initial 8-week period prior to the corrosion experiment. The growth of such a passive film was studied previously, e.g., [27], and its composition can be considered as Fe^II^_1−x_Fe^III^_2_O_4−x_ [28]. If x = 0, the film is similar to magnetite Fe_3_O_4_, and if x = 1, the film is similar to maghemite *γ*-Fe_2_O_3_.

When the temperature of the system reaches 80 °C, the passive film becomes unstable. The instability of the passive state for carbon steel in this specific cement grout is then not specifically linked to the Cl^−^ species present in the more aggressive solution used for the preliminary study because it was also observed in this work. To obtain further information, pH measurements were performed in the cement grout at 80 °C. It was observed that the increase of temperature led to a decrease of pH, from 10.7 at RT to 9.5 at 80 °C. This decrease of pH may be detrimental for the passive layer. Because the corrosion process that takes place subsequently is mainly associated with the formation of iron sulfides, it can be forwarded that the instability of the passive film results from the decrease of pH, the presence of sulfide species, or the combination of both factors.

Actually, the increase of temperature from RT to 80 °C and the resulting decrease of pH have necessarily an influence on the solubility of Fe(III) and Fe(II) compounds involved in the process. In the considered pH range, the decrease in pH should lead to an increase of solubility for Fe(II) and a decrease of solubility for Fe(III) [27]. This could lead to a release in solution of Fe^2+^ ions from the passive Fe^II^_1−x_Fe^III^_2_O_4−x_ film. In the presence of S(−II), these Fe^2+^ ions would then precipitate to form mackinawite according to the following reaction:

(2) $${\mathrm{Fe}}^{2+}+{\mathrm{HS}}^{-}\to \mathrm{FeS}+H^{+}$$

It can be noted that the formation of iron sulfide on steel in a basic medium has recently been observed [29], and the authors proposed a mechanism for the breakdown of the passive film associated with the sulfide species. In our case, the sulfide species present in the grout more likely come from the CEM-III cement but may also come from bentonite that can contain pyrite FeS_2_. Sulfide-producing microorganisms may also be involved, as the cement grout is rich in sulfate. However, the grout does not contain organic matter to sustain bacterial activity and growth. A bacterial origin for the sulfide species is then unlikely but cannot be completely discarded. Whatever their origin, the S(-II) dissolved species present in the grout definitely favored the formation of a corrosion product layer mainly composed of iron sulfides. These compounds are well-known corrosion products of steel and can form in a large variety of environments (e.g., [30,31,32,33,34,35,36,37,38]).

It is generally admitted that mackinawite FeS is the major phase that precipitates initially from the dissolved species [39]. It may transform with time because it is metastable with respect to various other phases [39]. It may also be oxidized even in anoxic conditions, a process that can lead to pyrite FeS_2_ [20,39] or to greigite via Fe(III)-containing mackinawite [20]. These two last compounds were clearly identified here. A third, unidentified compound was also detected. It may be an iron sulfide typical of alkaline conditions. Actually, most corrosion studies devoted to iron sulfides were performed in neutral or acidic conditions where H_2_S is involved (oil and gas industry) or in slightly alkaline conditions, such as those met in seawater where sulfide-producing bacteria have a strong influence (marine corrosion).

It is important to note that most iron sulfides are electronic conductors [40,41], including mackinawite FeS and greigite Fe_3_S_4_, which is structurally similar to magnetite. Magnetite is also an electronic conductor [42], and consequently all the corrosion products identified here are able to act as cathodic sites and can then favor corrosion cells.

In the absence of sulfide species, due to the high pH of the environment, magnetite (Fe_3_O_4_) would form instead of iron sulfides [43]. In the first preliminary study [15], magnetite was observed to be predominant, mackinawite FeS being observed as a minor phase. In the present case, magnetite was the minor phase with respect to the various iron sulfides. The SEM cross-section analysis (Figure 8) shows that the iron oxide (i.e., magnetite) is mainly present close to the steel surface, in an inner stratum. It forms a discontinuous 5-µm-thick layer and is mixed with iron sulfides; it is then clearly different from a passive layer that is a few nm thick. This preferential formation of magnetite in the inner stratum suggests that the sulfide concentration may be lower at the vicinity of the steel surface. Two reasons for this possible gradient of concentration can be forwarded: sulfide species (i) have to migrate from the environment towards the steel surface and (ii) may react with dissolved Fe(II) species farther from the steel surface to form FeS. The corrosion rates measured in the aerated conditions of the first study were much higher (up to 250 µm yr^−1^ observed during the 1-month experiment [15]). Because sulfide is a minor species in the grout, it can be forwarded that if the corrosion process produces a large amount of dissolved iron, the amount of available dissolved sulfide species is not sufficient for all the Fe^2+^ ions to precipitate as mackinawite. High corrosion rates would then favor the formation of magnetite, which is consistent with the results obtained here and in [15]. It is also possible that the aerated conditions considered in [15] favored the formation of magnetite. In any case, these new results clearly confirm the prominent influence of the sulfide species present in the grout.

In the present study, the average corrosion rate was kept low (3–6 µm yr^−1^) which may be the consequence of both anoxic conditions and protectiveness of the corrosion product layer. However, localized degradations were observed and associated with a significantly higher corrosion rate. In the most corroded areas, the measured corrosion depth reached 10 µm (for a 6-month experiment). In the preliminary study [15], localized corrosion was also observed, but only when the corrosion product layer was heterogeneous, i.e., mainly composed of magnetite with scattered areas of mackinawite. In a particular experiment, where FeS did not form and the steel electrode surface was covered by a homogeneous layer of Fe_3_O_4_, the corrosion process was uniform. The link between localized corrosion and heterogeneous FeS/Fe_3_O_4_ layers was also highlighted by other studies [30,31]. As already recalled above, Fe_3_O_4_ and FeS are electronic conductors that can favor galvanic effects and corrosion cells. In previous studies [15,30], the scattered areas of FeS corresponded to anodic zones surrounded by larger zones covered with magnetite that acted as the cathode where dissolved O_2_ reduction took place.

In the present case, the corrosion product layer is, conversely, mainly composed of iron sulfides (FeS, Fe_3_S_4_, and a third unidentified compound), and it is questionable whether the zones covered by magnetite are anodic regions. Moreover, anoxic conditions prevail here implying that the main cathodic reaction is water reduction. In aerated conditions, the cathodic process may be controlled by mass transport because dissolved O_2_ must diffuse in the electrolyte to reach a conductive surface. The cathodic process can then take place preferentially at the outer surface of a conductive layer of corrosion products (e.g., Fe_3_O_4_, Fe_3_S_4_, or FeS) because O_2_ does not need to diffuse through the pores of this layer. In the case of water reduction, mass transport has no influence. Localized corrosion may then rather be associated with the heterogeneity of the surrounding medium. Ca-, Al-, and Si-based particles coming from the cement grout were found close to the metal surface, incorporated in the corrosion product layer. They may for instance contribute to local variations of electrolyte composition and pH. The low amount of available sulfide species (coming from minor components of the grout) would also explain that in some regions of the steel/medium interface the local sulfide concentration was too low for FeS to precipitate, thus leading to magnetite instead. Finally, the resulting heterogeneity of the corrosion product layer would in turn favor the persistence of corrosion cells, due to the conductive properties of the obtained corrosion products.

## 5. Conclusions

The corrosion of carbon steel coupons buried in anoxic conditions at 80 °C in a specific low-pH cement grout designed for high-level radioactive waste disposal was studied via 6-month experiments. The solution used to saturate the cement grout simulated the pore water of the fresh grout. Despite alkaline conditions that should favor the formation of magnetite, or even that of a passive layer as Cl^−^ concentration was low, the corrosion process mainly led to the formation of iron sulfides (mackinawite, greigite, and a compound not yet identified). This result definitely confirms the prominent role of the sulfide species present in this grout.

This result also demonstrates that the passive state of the steel in the grout, established at RT [15], does not persist at 80 °C in anoxic conditions even if the Cl^−^ concentration of the pore solution is low.

Ca-, Si-, and Al-based components of the cement grout were also detected locally in the inner part, close to the metal, of the corrosion product layer. Magnetite was also identified in any case. The corrosion product layer can then be described as a heterogeneous layer mainly made of iron sulfides with scattered zones containing magnetite and components of the grout at the metal/cement grout interface.

The average corrosion rate proved low, between 3 and 6 µm yr^−1^, because of anoxic conditions and the protectiveness of the corrosion product layer. The term “low” is of course subjective. It must be linked to the technical role of the considered device. In the present case, the steel casing must keep its mechanical integrity for 500 years. Its thickness is planned to be ≥25 mm and a corrosion rate of 5 µm yr^−1^ would lead to a thickness loss of 2.5 mm after 500 years, i.e., only ≤10% of the overall thickness. From that perspective, 5 µm yr^−1^ can indeed be considered as a “low” corrosion rate.

However, optical profilometry measurements showed the presence of localized degradations on the surface of the carbon steel samples. These degradations reached, after 6 months of experiment, a depth of 10 µm. It is forwarded, as already reported in previous studies [15,30,31], that the heterogeneous corrosion product layer, mainly composed of conductive compounds (FeS, Fe_3_S_4_, and Fe_3_O_4_), can favor the persistence of corrosion cells and thus lead to locally aggravated degradations of the metal surface. Longer-term experiments (3 years) are in progress to study the evolution of these localized corrosion processes.

Finally, according to these conclusions, new cement grouts are now envisioned for the aimed application; they would have a slightly higher pH and a sulfide concentration as low as possible to avoid the local degradations observed with the specific grout considered here. Actually, pH is a key point. It must not be too high to mitigate the degradation of the glass matrix (typically, the pH 13 of common Portland cement must be avoided), but it has to be sufficiently high to allow the passivation of carbon steel.

## Figures and Tables

**Table 1:** Composition (wt.%) of API 5L X65 carbon steel.

Fe	C	Si	Mn	P	S	Cr	Mo	Ni	Cu	Nb	Ti	V
98.09	0.041	0.33	1.33	0.009	0.002	0.056	0.015	0041	0.029	0.042	0.001	<0.005

**Table 2:** Preparation of the cement grout: constituents used and corresponding information.

Constituent	Reference/Commercial Designation	Information Can Be Obtained at the Following URL
Cement	CEM III/C 32,5 N SR CE PM NF	https://uploads.gedimat.fr/DOCUMENT/TYPE1/0000024691833.pdf (accessed on 15 June 2021)
Silica fume	S95 DM	http://www.condensil.com/fre_FR/fumee-de-silice (accessed on 15 June 2021)
Bentonite	2 HS 1	http://www.strainelectric.com/wp-content/uploads/2016/03/SDS-Bentonite-Sodium-SDS-12-01INT13-09.pdf (accessed on 15 June 2021)
Hydrotalcite	MG 70-PURAL	http://www.sasoltechdata.com/tds/PURAL-MG.pdf (accessed on 15 June 2021)
Water	Evian^®^	https://www.evian.com/fr/notre-eau/caracteristiques-de-leau-devian/ (accessed on 15 June 2021)

**Table 3:** Composition of the solution used for the corrosion experiment.

Constituent	Concentration in Solution (mol L^−1^)
K_2_SO_4_	0.025
NaHCO_3_	0.0125
CaSO_4_·2H_2_O	0.008
Na_2_SiO_3_·5H_2_O	0.0029
Al_2_(SO_4_)_3_·18H_2_O	2 × 10^−4^
KCl	10^−5^

**Table 4:** Proportion (wt.%) of the elements identified during the analysis at spots 1, 2, and 3.

Analyzed Zone	O	Na	Mg	Al	Si	P	S	K	Ca	Mn	Fe	Total
Spot 1	12.6	0.1	1.9	0.4	12.0	0.1	21.6	-	2.0	0.5	48.8	100
Spot 2	3.0	-	-	-	0.5	0.1	33.7	-	0.9	0.2	61.6	100
Spot 3	9.4	-	1.9	0.4	11.1	0.1	24.1	0.1	1.9	0.6	50.4	100

## Data Availability

The data presented in this study are available on request from the corresponding author.
